# Evaluation of a large-scale aptamer proteomics platform among patients with kidney failure on dialysis

**DOI:** 10.1371/journal.pone.0293945

**Published:** 2023-12-11

**Authors:** Yue Ren, Peifeng Ruan, Mark Segal, Mirela Dobre, Jeffrey R. Schelling, Upasana Banerjee, Tariq Shafi, Peter Ganz, Ruth F. Dubin, the CRIC Study Investigators

**Affiliations:** 1 Department of Biostatistics, Epidemiology, and Informatics, Perelman School of Medicine, University of Pennsylvania, Philadelphia, Pennsylvania, United States of America; 2 Peter O’Donnell Jr School of Public Health, UT Southwestern, Dallas, Texas, United States of America; 3 Department of Epidemiology and Biostatistics, University of California, San Francisco, San Francisco, California, United States of America; 4 Division of Nephrology and Hypertension, University Hospitals Cleveland Medical Center, Cleveland, Ohio, United States of America; 5 Department of Physiology & Biophysics, Case Western Reserve University of School of Medicine, Cleveland, Ohio, United States of America; 6 Department of Internal Medicine, Hurley Medical Center/Michigan State University, Flint, Michigan, United States of America; 7 Division of Kidney Diseases, Hypertension and Transplantation, Houston Methodist Hospital, Houston, Texas, United States of America; 8 Division of Cardiology, University of California, San Francisco, San Francisco, California, United States of America; 9 Division of Nephrology, University of Texas Southwestern Medical Center, Dallas, Texas, United States of America; Kindai University Faculty of Medicine, JAPAN

## Abstract

**Background:**

Patients with kidney failure suffer high mortality, and we currently lack markers for risk stratification for these patients. We carried out a quality control study of a modified aptamer assay (SomaScan v.4.0) that measures ~ 5000 proteins, in preparation for a larger study using this platform in cohorts with kidney failure.

**Methods:**

Forty participants from the Cardiac, Endothelial Function and Arterial Stiffness in End-Stage Renal Disease (CERES study) were selected to analyze technical and short-term biological variability, orthogonal correlations and differential protein expression in plasma from patients who died during 2.5 year follow-up. Long-term (one year) variability was studied in 421 participants in the Chronic Renal Insufficiency Cohort. We evaluated 4849 aptamers (4607 unique proteins) using data formats including raw data and data formatted using Adaptive Normalization by Maximum Likelihood (ANML), an algorithm developed for SomaScan data in individuals with normal kidney function.

**Results:**

In ANML format, median[IQR] intra-assay coefficient of variation (CV) was 2.38%[1.76, 3.40] and inter-assay CV was 7.38%[4.61, 13.12]. Short-term within-subject CV was 5.76% [3.35, 9.72]; long-term CV was 8.71%[5.91, 13.37]. Spearman correlations between aptamer and traditional assays for PTH, NT-proBNP, FGF-23 and CRP were all > 0.7. Fold-change (FC) in protein levels among non-survivors, significant after Bonferroni correction, included SVEP1 (FC[95% CI] 2.14 [1.62, 2.82]), keratocan (1.74 [1.40, 2.15]) and LanC-like protein 1 (0.56 [0.45, 0.70]). Compared to raw aptamer data, technical and short-term biological variability in paired samples was lower in ANML-formatted data. ANML formatting had minimal impact on orthogonal correlations with traditional assays or the associations of proteins with the phenotype of mortality.

**Conclusions:**

SomaScan had excellent technical variability and low within-subject short-term variability. ANML formatting could facilitate comparison of biomarker results with other studies that utilize this format. We expect SomaScan to provide novel and reproducible information in patients with kidney failure on dialysis.

## Introduction

Patients with kidney failure on dialysis suffer extraordinarily high rates of mortality at ~18% per year, with 50% of deaths attributed to cardiovascular disease (CVD) [1]: namely, atherosclerotic CVD, heart failure, and sudden cardiac death. Over the past decade, the number of patients with kidney failure has increased [2], and Medicare spending for these patients has almost doubled [3]. We currently lack viable CVD markers for risk stratification for patients with kidney failure; traditional CVD risk factors, such as hypercholesterolemia and obesity, have reverse associations with CVD outcomes in the kidney failure population [4]. Kidney Disease Improving Global Outcomes recently cited the need for “development and validation of kidney failure-specific CVD risk prediction scores [5].” We need new biomarkers not only for risk stratification but also for a better understanding of the biology underlying accelerated CVD in this population.

Circulating protein levels can serve as modifiable biomarkers for CVD risk, guide therapy and elucidate biological pathways and potential therapeutic targets [6–8]. Biomarker research for patients with kidney failure on dialysis presents several challenges, including the high concentration of some proteins due to low glomerular filtration rate and potential interference of uremic solutes. Additionally, hemodialysis causes hemoconcentration due to removal of intravascular volume and some solutes <50kDa. Apart from these physiological factors, there are also analytical considerations. SomaScan, an advanced modified aptamer assay that measures ~ 5000 proteins in just 55μl of plasma with high sensitivity and specificity, has the potential to provide a more comprehensive evaluation of potential protein biomarkers, compared to prior studies examining fewer proteins [9]. Data from SomaScan is reported in relative fluorescence units(RFU), and is routinely standardized using Adaptive Normalization by Maximum Likelihood (ANML), an algorithm that reduces the range of outliers in experimental datasets by conforming to aptamer values in the Covance patient dataset of relatively healthy individuals [10]. Any protein level in a patient with kidney failure could be elevated by 2–10 -fold compared to a healthy individual, depending on the physiological processes affecting the protein. ANML algorithms are applied to all protein measures, regardless of protein concentration, but it is not known whether the ANML format could disproportionately affect proteins at very high or low concentrations and thus potentially bias association studies among participants with kidney failure towards the null. We designed our study to provide a comparison of ANML, median normalized, and raw formatted data in the kidney failure population.

We conducted a quality control study of SomaScan (Somalogic, Boulder, Colorado) in 40 participants selected from the Cardiac, Endothelial Function and Arterial Stiffness in End-Stage Renal Disease (CERES) study, a cohort of 200 patients with kidney failure on hemodialysis who were followed for median of 2.5 years for clinical outcomes including all-cause mortality. We sought to demonstrate technical intra-and inter-assay variability and short-term within-subject variability of the SomaScan assay. Additionally, we extracted long-term within-subject variability from an ongoing study of individuals on hemodialysis participating in the Chronic Renal Insufficiency Cohort (CRIC). Using pilot samples in CERES, we characterized the proteins that saturate in the assay, or in other words, proteins present at higher concentrations than what is targeted in the current SomaScan assay. We analyzed preliminary orthogonal correlations of several aptamer measures with traditional marker assays. We explored the impact of two data standardization algorithms, median normalization and ANML, on variability metrics, orthogonal correlations and fold change analysis of protein levels in CERES participants who died vs. those who survived.

## Materials and methods

### Sources for plasma samples

Forty participants on hemodialysis were selected from the CERES study for proteomics assays. CERES was a prospective observational study of 200 patients on hemodialysis or peritoneal dialysis, designed to provide comprehensive assessment of cardiac mechanics with modern echocardiographic measures and to determine the utility of these measures for predicting cardiovascular outcomes [11–13]. CERES participants were recruited from the University of California San Francisco (UCSF) Kidney Pancreas Transplant Clinic, the Zuckerberg San Francisco General Hospital Chronic Dialysis Unit, and five Bay Area Fresenius and DaVita dialysis units between May 28, 2013 and April 20, 2016. To be included, patients had to be on hemo- or peritoneal dialysis for at least 1 month. Study visits occurred at the Zuckerberg San Francisco General Clinical Research unit, and patients on hemodialysis came in the morning after the first hemodialysis session of the week. Participants were instructed to take all medications, have no caffeine or tobacco 24 hours prior to the visit, and have a light meal prior to the study visit. Blood samples were centrifuged at 1100 g for 10 minutes in a refrigerated centrifuge within 30 minutes of phlebotomy, and plasma was frozen at -80°C. Cause of death was adjudicated by review of electronic records available for patients hospitalized at UCSF or Zuckerberg San Francisco General, or by review of notes in the UCSF kidney transplant waitlist database. The 40 patients selected for this pilot study included at least 1/3 women, and 1/3 participants who experienced a cardiovascular event or died during the median follow-up period of 2.5 years. These included 5 participants on hemodialysis with short-term follow-up plasma samples, collected 1–4 weeks apart on the day after the first dialysis session of the week. A separate group of 15 CERES participants were chosen for inter-assay variability based on the amount of available frozen plasma, and samples from these participants were split into pairs at UCSF, and then sent to different sites, from where they were sent as blinded controls among the boxes shipped to Somalogic for the larger study that included CRIC participants. Patients gave written informed consent, and the study protocol was approved by the UCSF Committee for Human Research. As the Principal Investigator, Dr. Dubin personally enrolled patients into the study, and therefore had access to identifiable information for some participants. No identifiable information was permanently stored in the CERES database.

Plasma samples from 421 CRIC participants were assayed for proteomics as part of a new study of large-scale proteomics among patients on hemodialysis. CRIC enrollment and exclusion criteria are detailed elsewhere [14,15]. CRIC participants had progressed to kidney failure and started hemodialysis; samples were drawn during the years (2005–2018) across 13 sites. The CRIC study protocols adhered to ethics regulations of each institution where participants were enrolled, requiring approval from the following committees: University of Pennsylvania Institutional Review Board, Federalwide Assurance # 00004028; Johns Hopkins Institutional Review Board NA_00044034 / CIR00004697; The University of Maryland, Baltimore Institutional Review Board; University Hospitals Cleveland Medical Center Institutional Review Board; MetroHealth Institutional Review Board; Cleveland Clinic Foundation Institutional Review Board IRB #5969; University of Michigan Medical School Institutional Review Board; Wayne State University Institutional Review Board; University of Illinois at Chicago Institutional Review Board; Tulane Human Research Protection Office, Institutional Review Boards, Biomedical Social Behavioral, IRB #140987; Kaiser Permanente Northern California Institutional Review Board. The Atherosclerosis Risk in Communities (ARIC) Study adhered to ethics regulations from and was approved by a single Institutional Review Board (sIRB) at Johns Hopkins School of Medicine (FWA00005752; IRB00311861) and Institutional Review Boards (IRB) at all participating institutions: University of North Carolina at Chapel Hill, Johns Hopkins University School of Public Health, University of Minnesota, Wake Forest University Health Sciences, University of Mississippi Medical Center, Baylor College of Medicine, University of Texas Houston Health Science Center, and Brigham and Women’s Hospital. Study participants provided written informed consent at all study visits. All study participants provided written informed consent. CRIC samples were drawn on a non-dialysis day in 80% of patients; the rest were drawn directly prior to dialysis. For any given site, the timing was consistent for each of the two blood samples for a given participant. Participants had proteomics measured at study baseline, which occurred after initiation of dialysis, and then at a second timepoint median[IQR] 0.98 years [0.83, 1.11] later. Participants did not fast and blood was promptly centrifuged and sent on dry ice to the Central Lab at University of Pennsylvania where it was aliquoted and stored at -80°C.

### SomaScan assay

SomaScan V4.0 is an assay based on modified aptamers, which are chemically modified single strands of deoxyribonucleic acid ~40 nucleotides long, as binding reagents for target proteins [7,8,10,16–18]. Modified aptamers bind to proteins with high affinity similar to antibodies (lower limit of detection 10^−15^ moles per liter.) [8,16,17] “Pull-down” studies, in which the aptamer-protein complexes were isolated and the identities of the bound proteins were verified by targeted mass spectrometry and gel electrophoresis, have been performed for 920 proteins among 1305 proteins in a previous version of the assay [18]. These studies showed that > 95% of aptamers correctly targeted the intended proteins (for those proteins in concentrations sufficient to be detected by mass spectrometry). The samples on the SomaScan assay are run at three different dilutions to assay each analyte within its linear range of concentrations. The assay results are quantified on a hybridization microarray and reported in RFU. SomaLogic has procedures for data calibration, standardization and internal controls, typical of microarray technologies. After running the assay, SomaLogic standardizes the entire protein dataset using ANML to remove bias in the assay. This is an iterative procedure that standardizes values in the experimental dataset according to expected measurements from a reference distribution derived from a cohort of relatively healthy individuals recruited by Covance [10]. Efficacy of standardization is demonstrated by a reduction in the variation in replicate samples. Median normalization is an alternative to ANML that utilizes the median value of each protein in the dataset as the reference to standardize the dataset, rather than an external reference. Further details on the assay procedures and data formatting are found in S1 SomaScan technical information.

In this study, CERES samples were run on SomaScan V4.0, a platform with a total of 5284 aptamers (4746 unique proteins). After excluding 305 aptamers targeting non-human proteins, and 130 investigational aptamers, V4 is comprised of 4849 aptamers (4607 unique proteins). CRIC samples and the 15 inter-assay pairs were run on SomaScan V4.1, with a total of 7596 aptamers (6432 unique proteins), but for this study we restricted our variability analyses to proteins that were included in V4 in order to compare variability metrics. As part of our pilot study in CERES, Somalogic reported that 47 proteins ‘saturated’ in the assay, meaning that they were present at concentrations too high to be measured accurately (>80,000 RFU in > half of the forty CERES samples). After excluding non-human proteins, investigational proteins and saturated proteins, variability analyses in CERES and CRIC included 4802 proteins (4564 unique proteins). We did not exclude saturate proteins in our screen for proteins associated with mortality. Proteins excluded from the variability analyses are listed in **S2 Table 1 in** S2 File.

### Traditional assays

For CERES participants, calcium, phosphorus, albumin and parathyroid hormone (PTH)were drawn the day of the study visit and processed at the University of Maryland laboratory using Siemens Dimension Vista system with Flex reagent cartridges (Siemens Healthcare, Newark, DE) High sensitivity cardiac Troponin T (hsTnT) and N-terminal pro B-type natriuretic peptide (NT-proBNP) measurements were performed on the e 601 cobas analyzer (Roche Diagnostics, Indianapolis, Indiana). The coefficient of variation (CV) for the NT-proBNP assay was 2% to 5% during the testing period, with an analytical measurement range 5–35,000 ng/L. High sensitivity C-reactive protein (CRP) measurements were performed on the Dimension Vista (Siemens Healthcare, Newark, DE). The hsCRP values associated with CVD risk are as follows: low risk < 1.0 mg/L; average risk 1.0–3.0 mg/L; high risk > 3.0 mg/L [19]. Precision of the assay was <5.2% across the measurement range. Fibroblast growth factor-23(FGF-23) was assayed with Immunotopics 2^nd^ Generation Enzyme-Linked ImmunoSorbent Assay for human intact FGF-23 (Immunotopics Inc., San Clemente, CA); it does not measure N-terminal or C-terminal fragments. The manufacturer reports intra-assay CV of 2.0% and inter-assay CV of 3.5%. Parathyroid hormone (PTH) was measured using the Elecsys 2010 PTH Immunoassay (Roche Diagnostics, Indianapolis, Indiana), which has inter- and intra-assays CVs ≤ 3%. Troponin I was measured using a high-sensitivity Troponin I assay (Siemens Healthcare, Newark, DE).

### Analytical methods

Clinical characteristics for CERES participants were summarized as median [interquartile range] [IQR] for skewed or mean (standard deviation) (SD) for parametric continuous variables. Clinical factors were compared between survivors and non-survivors using chi^2^ for categorical variables or Wilcoxon rank sum for continuous variables. Our first step for evaluating variability in technical duplicates (intra-assay and inter-assay variability) was to perform hierarchical clustering by Euclidean distances. For each type of variability parameter, we calculated the CV (SD/mean) and spearman *rho* correlation between the same protein measured in the pair. Median [IQR] CV was calculated for each protein among pairs analyzed, and for aggregate results, we report the median [IQR] CV among all proteins. For analysis of orthogonal correlations between aptamer and traditional measures, the aptamer measure was averaged in the duplicate pair. Correlations for CRP, FGF23, NT-proBNP, troponin T, troponin I and PTH were assessed using scatterplots and Spearman’s *rho*. For the analysis of protein level fold change between survivors and non-survivors, we transformed protein RFU using log_2_. Statistical inference surrounding fold change and differential protein expression was performed using *limma*, an R/Bioconductor software package that provides an integrated approach to analyzing data from a variety of study designs [20]. Limma facilitates simultaneous comparison between numerous targets (here 4849 proteins). It achieves analytic stability even in settings with small numbers of subjects by borrowing information among proteins using empirical Bayes methods. Although we designated p<1x10^-5^ as a Bonferroni-corrected significant fold change, we also considered proteins meeting the criterion of false discovery rate (FDR) q-value <0.05. From among proteins with fold change significant at FDR q <0.05, we present 10 proteins with the highest fold change and 10 with the lowest fold change. The adjusted model for fold change includes variables found to be associated with mortality in CERES participants at p<0.05 (age, history of stroke, systolic blood pressure, albumin, phosphorus) and also dialysis modality (hemodialysis vs peritoneal dialysis) since dialysis modality or higher residual urine output typical for peritoneal dialysis patients could influence protein levels. For variability analyses, orthogonal correlations and fold change, we analyzed RFU data in 3 formats: raw data, median normalized, and ANML. These data formats were provided by Somalogic. Our analyses of CERES and CRIC data were performed using R version 4.2.2.

## Results

### Study participants

Among CERES participants, average age was 55 years, 40% were female, and 75% were of non-white race. Fifty-five percent had a history of diabetes, 33% history of CVD, and systolic blood pressure was 138 mm Hg. Median time since dialysis initiation was 40 months, 6 were on peritoneal dialysis and 34 on hemodialysis. (Table 1) Median [IQR] follow-up time was 31 [13, 40] months, and among the 14 CERES participants who died, 29% died of cardiovascular causes, 21% infection or malignancy, and 50% of unknown causes. Among CRIC participants, distributions of age, gender and race were similar to CERES, but CRIC participants had higher rates of diabetes and CVD, and shorter time since dialysis initiation (median[IQR] 7.56 months [4.04, 11.50]). (**S2 Table 2 in** S2 File).

**Table 1:** Baseline characteristics of 40 participants with kidney failure.

Characteristic	All participants N = 40	Survivors N = 26	Non-survivors N = 14	p-value
Age (years)	55.0 (12.7)	51.1(13.2)	60.5(9.5)	**0.037**
Male gender	24(60%)	8(57%)	16(62%)	0.79
Race				
White	10(25%)	6(23%)	4(29%)	0.70
Non-white	30(75%)			
Time on dialysis(months)	40(21, 68)	53(53)	72(95)	0.64
Diabetes	21 (53%)	13(50%)	8(57%)	0.66
History of myocardial infarction	4 (10%)	5(19%)	6(43%)	0.66
History of heart failure	6(15%)	4(15%)	2(14%)	0.93
History of stroke	5(12.5%)	1(3.9%)	4(29%)	**0.024**
Peritoneal dialysis vs hemodialysis	6 (15%)	5(19%)	1(7%)	0.31
Fistula or graft access	30(75%)	20(77%)	10(71%)	0.70
Cause of renal failure				0.60
Diabetes	15(38%)	9(35%)	6(43%)	
Hypertension	9(23%)	5(19%)	4(29%)	
Glomerulonephritis	2(5%)	1(4%)	1(7%)	
Other	11(27.5%)	11(42%)	3(21%)	
Anuric	28(70%)	17(65%)	11(78%)	0.39
SBP (mmHg)	138 (24)	131(23)	151(21)	**0.025**
DBP (mmHg)	76 (15)	75(64, 85)	71(64, 87)	0.78
BMI (kg/m^2^)	29 (24, 34)	27 (24, 31)	33 (24, 34)	0.23
HDL (mg/dL)	44(15)	42(37, 50)	33(29, 52)	0.22
LDL (mg/dL)	69(52.5, 100)	73(53, 106)	64(46, 88)	0.33
Calcium (mg/dL)	8.7 (0.79)	8.6(8.1, 9.2)	8.6(7.7, 9.1)	0.50
Phosphorus (mg/dL)	4.6 (1.28)	4.5(4, 5.7)	3.8(3.5, 4.8)	**0.037**
Parathyroid hormone (pg/ml)	312(196, 460)	322(240, 473)	231(146, 407)	0.16
Albumin (g/dL)	3.6(0.49)	3.7(3.4, 4.1)	3.3(3, 3.6)	**0.010**
Kt/V	1.59 (0.24)	1.5(1.4, 1.7)	1.6(1.5, 1.8)	0.60
Hemoglobin (g/dL)	11.1 (1.3)	11.2(10.8, 11.7)	10.8(9.7, 11.7)	0.15
Follow-up (months)	31(12.5, 40)	39(30, 46)	9(5, 19)	**0.001**

P-values based on Wilcoxon rank sum for continuous variables and chi2 for categorical variables.

### Sample quality and assay saturation

Somalogic performed blinded clustering of samples and found only one that could not be accurately paired to participant ID, and we excluded this one sample from our analysis. Somalogic also reported finding 79 aptamers with signal > 80,000 RFU, that were saturated in the SomaScan assay. Only 47 of these saturated in more than half of samples analyzed using ANML data. (**S2 Table 1 in** S2 File) Among 842 CRIC samples, there were 61(7%) samples that were flagged and 32 proteins that saturated in > ½ of samples using ANML data.

### Technical variability

**Fig 1:**
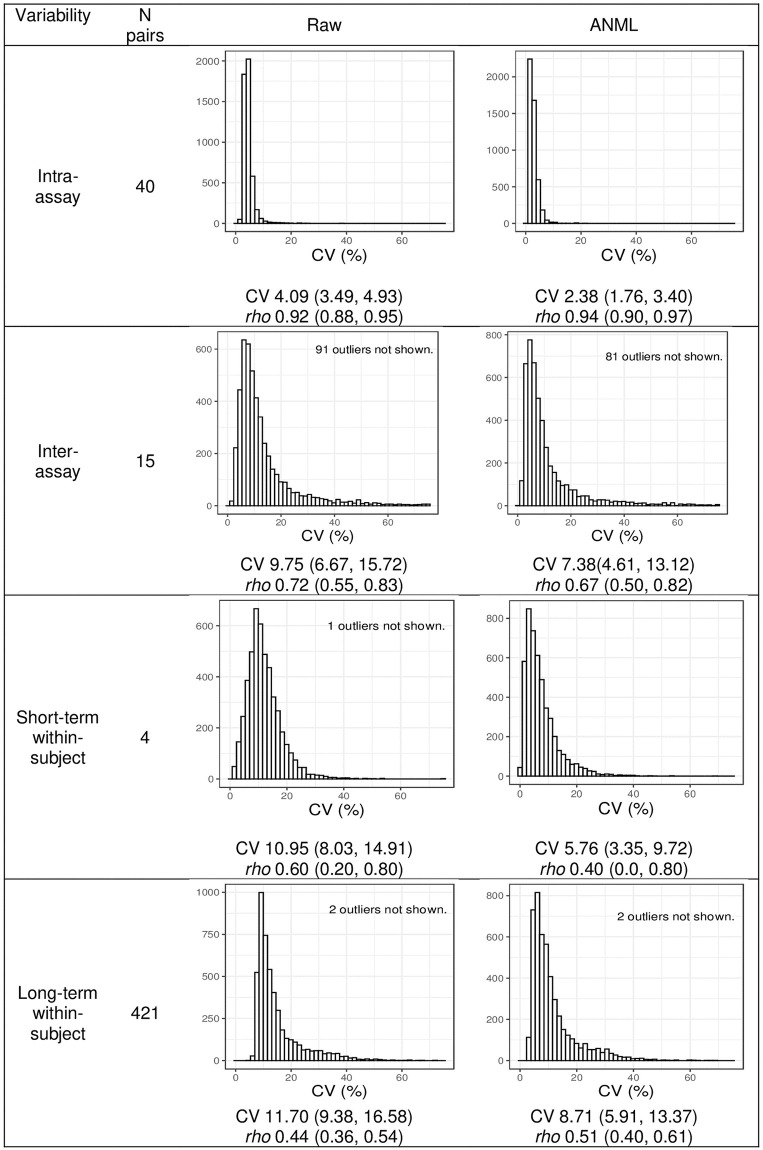
Technical and biological variability of somascan in plasma samples of patients with kidney failure. Coefficient of variation (CV) and spearman *rho* correlations between pairs of samples are shown above. Data are from CERES study with the exception of long-term within-subject variability, calculated from CRIC data.

Intra-assay variability was evaluated in 40 CERES sample duplicates run on the same plate. Using raw data, median [IQR] intra-assay CV was 4.1% [3.5, 4.9] and spearman *rho* (95%CI) was 0.92(0.88, 0.95). Using median normalized data, median [IQR] intra-assay CV was 2.3% [1.8, 3.2], and spearman *rho* was 0.94 (0.90, 0.97). Using ANML data, median [IQR] CV was 2.38%[1.76, 3.40], and spearman *rho* 0.94 (0.90, 0.97). ANML and median normalized data exhibited very similar variability metrics, and henceforth we will describe only raw and ANML CVs. Aggregate CV and correlation analyses are illustrated in Fig 1. Eight (0.2%) proteins had intra-assay CV>20% in raw format, and 6 (0.1%) had CV>20% in ANML format. Proteins with intra-assay CV>20% are listed in **S2 Table 3 in** S2 File.

Inter-assay variability in was evaluated in 15 CERES samples that were assayed approximately 1 month apart, with each member of the pair prepared and sent from a different site. Using raw data, median [IQR] CV was 9.8% [6.7, 15.7], and spearman *rho* was 0.7 (0.6, 0.8). Using ANML data median [IQR] CV was 7.4% [4.6, 13.1], and spearman *rho* was 0.7 (0.50, 0.8). 301 (6.3%) proteins had CV>40% in raw format, and 261 (5.4%) had CV>40% in ANML format. (**S2 Table 4 in** S2 File)

### Biological variability

Short-term within-subject variability was measured in 5 CERES participants on hemodialysis, in samples drawn 1–4 weeks apart. A sample in one interval pair was flagged by Somalogic for sample quality and in our own blinded analysis, this one sample did not cluster with the other sample from this participant. We excluded this pair, leaving 4 participants with pairs of samples to analyze for short-term variability. For two participants the time interval between samples was 7 days; for 1 participant, the time interval was 3 weeks and for one participant it was 4 weeks. Among the 4 participants, using raw data, median [IQR] CV was 11% [8.0, 15], and spearman *rho* (95%CI) was 0.60 (0.20, 0.80). Using ANML data, median [IQR] CV was 5.8% [3.4, 9.7], and spearman *rho* (95%CI) was 0.4 (0.0, 0.80). Samples from the participant with 4 weeks between samples had the highest CV (raw format: 20.9% [11.1, 31.2]; ANML: 10.97% [4.95, 21.09]). CVs and *rho* correlations for each participant are listed in **S2 Table 5 in** S2 File.

Long-term variability was measured in 421 CRIC participants on hemodialysis who had samples drawn median [IQR] 0.98 [0.83, 1.11] years apart. Using raw data, median [IQR] CV was 11.7%[9.4, 16.6], and spearman *rho* (95%CI) was 0.4 (0.4, 0.5). Using ANML data, median [IQR] CV was 8.7% [5.9, 13.4], and spearman *rho* (95%CI) was 0.5 (0.4, 0.6). The time interval among participants was variable, so we calculated fold change per year: Raw median [IQR] fold change per year was 1.09 [1.07, 1.12] and ANML median [IQR] fold change per year 1.03 [1.01, 1.06]. Long-term CVs and fold change for all proteins are listed in **S2 Tables 6 and 7 in** S2 File, which show CVs calculated in raw data and ANML data, respectively.

**Fig 2:**
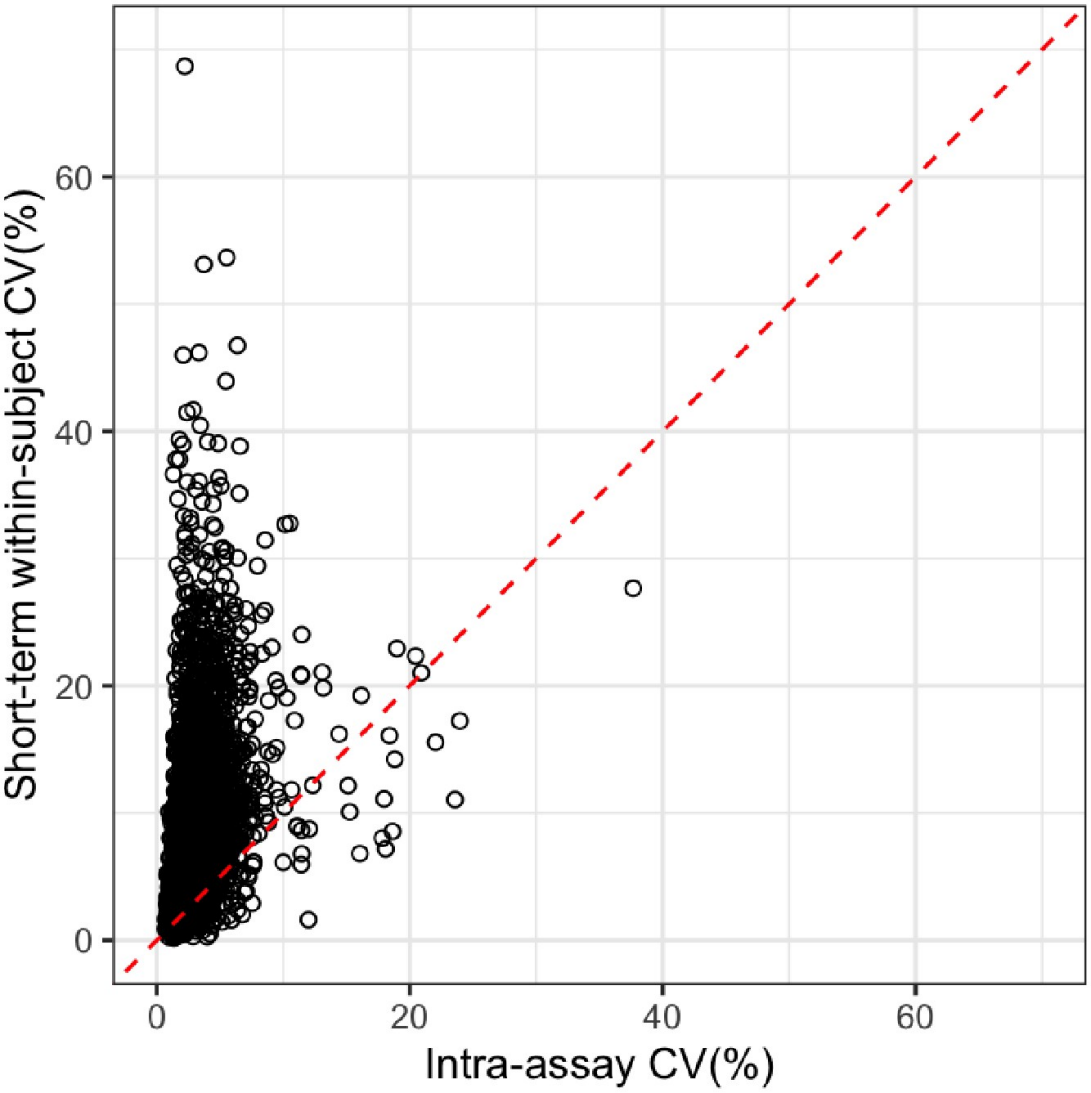
Biological and technical variability of SomaScan. Short-term biological and intra-assay CVs for 4802 proteins are shown in 4 study participants who had samples available for both types of CVs from the CERES cohort.

To compare analytical and biological variability, we compared intra-assay duplicate CVs to within-subject short-term variability CVs among 4 hemodialysis patients in CERES (Fig 2). In the ANML format, the intra-assay (split duplicate) CV was less than the short-term within-subject CV for 92% of proteins. We also tested the correlation between protein concentration (RFU) and either technical or biological CV in CERES samples, and found for RFU–technical CV *rho* = 0.57, and for RFU–short-term biological CV *rho* = 0.50.

### Orthogonal correlations

**Fig 3:**
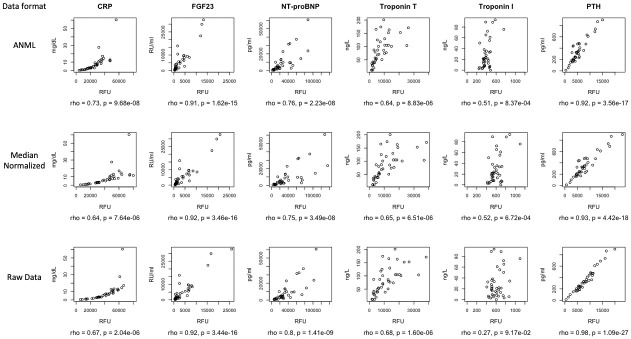
Orthogonal correlations for aptamer and traditional assays in CERES participants. Spearman *rho* correlations were analyzed for aptamer and traditional assays. Aptamer measures were analyzed in three data formats: raw data, median normalized, and ANML. X-axes show the aptamer measure in relative fluorescence units (RFU), and the y-axes show the traditional assay. CRP: c-reactive protein. FGF23: fibroblast growth factor 23. NT-proBNP: N-terminal pro-B-type natriuretic peptide. PTH: parathyroid hormone.

Orthogonal correlations between aptamers and traditional measures were examined in three data formats: raw, median normalized, and ANML. (Fig 3) The range of aptamer values is smaller for ANML data compared to raw data. However, spearman’s *rho* were virtually the same across three data formats for each marker. The markers with the highest correlations were CRP, FGF23, NT-proBNP, and PTH (*rho* > 0.7). For Troponin T, *rho* = 0.64; for Troponin I, *rho* = 0.51.

### Fold change

We analyzed the difference in protein level between 14 CERES participants who died and 26 participants who survived and expressed the difference as fold change. We carried out this analysis for three data formats to find whether statistical significance or effect size was impacted by ANML data formatting. First, we ranked all proteins by significance or effect size. At FDR q<0.05, there were 800 proteins significant in the raw format, 817 in median normalized format, and 875 in ANML format. At Bonferroni significance, there were 5 proteins in raw format, 8 proteins in median normalized, and 10 proteins in ANML format. While data formatting influenced the number of proteins reaching statistical significance, there was consistency in which proteins were selected at highest significance. For example, Sushi, von Willebrand factor type A, EGF and pentraxin domain containing 1 (SVEP1), keratocan, and LanC like glutathione S-transferase 1 (LANCL1) were significant after Bonferroni correction in all three data formats. (Table 2) Correlations of p-values between raw and ANML formats was only moderate (rho = 0.60), but correlations of effect sizes between raw and ANML formats was excellent (rho = 0.93.) (**S2 Table 8 in** S2 File) To illustrate the similarity of effect sizes across assays and data formats, fold change of NT-proBNP is shown using the traditional assay and across three aptamer data formats in Table 3.

**Table 2:** Proteins meeting significance criteria for fold change in CERES participants who died, using three normalization formats.

Data format	Fold change>1			Fold change<1		
	q<0.05	p<1x10-5		q<0.05	p<1x10-5	
	#	#	Protein names	#	#	Protein names
Raw	102	2	**SVEP1, keratocan**	699	3	IGF-1, IGFBP-2, **LANC-1**
Median normalized	286	6	**SVEP1**, alpha 2 macroglobulin, **keratocan**, fibulin-5, IGFBP-7, CD166 antigen	531	2	**LANC-1**, IGFB-5
ANML	188	2	**SVEP-1, keratocan**	687	8	IGF-1, ADH-6, **LANC-1**, transketolase, chloride intracellular channel protein 4, triosephosphate isomerase, programmed cell death 6-interacting protein, IGFB-5

Numbers of proteins meeting significance criteria at FDR <0.05 or Bonferroni p<0.05/5000 for fold-change in non-survivors, without adjustment for covariates. Bold denotes proteins meeting Bonferroni significance in all three data formats.

SVEP1: Sushi, von Willebrand factor type A, EGF and pentraxin domain-containing protein 1 IGFBP: Insulin-like growth factor-binding protein(s) IGF-1: Insulin-like growth factor 1 LANC-1: LanC-like protein 1 ADH-6: Alcohol dehydrogenase 6.

**Table 3:** Fold Change (FC) in protein levels among non-survivors compared to survivors in 40 CERES participants.

Protein	Unadjusted				Adjusted			
	FC	95% CI	p-value	q-value	FC	95% CI	p-value	q-value
	**Proteins with largest FC**							
**SVEP1**	**2.14**	**(1.62, 2.82)**	**2.08x10-6**	**0.0018**	1.44	(1.08, 1.92)	0.014	0.43
**Keratocan**	**1.74**	**(1.40, 2.15)**	**6.05x10-6**	**0.0021**	1.62	(1.22, 2.15)	0.0013	0.43
Adhesion G-protein coupled receptor G1	1.74	(1.38, 2.21)	1.9x10-5	0.0030	1.71	(1.26, 2.31)	0.0011	0.43
SLAM family member 6	1.44	(1.21, 1.71)	0.00012	0.0043	1.37	(1.10, 1.69)	0.0059	0.43
Alpha 2 macroglobulin	1.38	(1.18, 1.61)	0.00013	0.0042	1.36	(1.14, 1.63)	0.0011	0.43
Thrombospondin-2	2.25	(1.52, 3.32)	0.00014	0.0042	1.87	(1.15, 3.02)	0.013	0.43
Hepatocyte growth factor	1.48	(1.23, 1.79)	0.00015	0.0043	1.42	(1.11, 1.81)	0.0058	0.43
Heat shock 70kDaprotein	1.45	(1.21, 1.75)	0.00017	0.0044	1.39	(1.10, 1.76)	0.0062	0.43
Macrophage scavenger receptor types I and II	1.70	(1.30, 2.23)	0.00025	0.0051	1.47	(1.05, 2.06)	0.027	0.43
Carbohydrate sulfotransferase 9	1.57	(1.25, 1.97)	0.00025	0.0051	1.54	(1.15, 2.06)	0.0052	0.43
	**Proteins with smallest FC**							
**Transketolase**	**0.66**	**(0.56, 0.77)**	**3.65x10-6**	**0.0018**	0.73	(0.60, 0.89)	0.003	0.43
**Chloride intracellular channel protein 4**	**0.66**	**(0.55, 0.77)**	**6.75x10-6**	**0.0021**	0.73	(0.59, 0.90)	0.0048	0.43
**Triosephosphate isomerase**	**0.60**	**(0.50, 0.73)**	**3.66x10-6**	**0.0018**	0.69	(0.54, 0.87)	0.0032	0.43
**Programmed cell death 6-interacting protein**	**0.59**	**(0.48, 0.73)**	**8.33x10-6**	**0.0022**	0.67	(0.51, 0.87)	0.0037	0.43
**Alcohol dehydrogenase 6**	**0.58**	**(0.47, 0.71)**	**1.84x10-6**	**0.0018**	0.66	(0.51, 0.84)	0.0016	0.43
**LanC-like protein 1**	**0.56**	**(0.45, 0.70)**	**3.44x10-6**	**0.0018**	0.65	(0.50, 0.86)	0.0028	0.43
**Insulin-like growth factor-binding protein 5**	**0.55**	**(0.44, 0.70)**	**7.25x10-6**	**0.0021**	0.69	(0.53, 0.90)	0.0078	0.43
**Insulin-like growth factor I**	**0.52**	**(0.42, 0.65)**	**4.45x10-7**	**0.0013**	0.59	(0.45, 0.76)	0.00016	0.43
Coagulation Factor XI	0.75	(0.66, 0.84)	1.43x10-5	0.0030	0.78	(0.66, 0.91)	0.0020	0.43
Differentially expressed in FDCP 6 homolog	0.60	(0.49, 0.74)	1.46x10-5	0.0030	0.69	(0.53, 0.90)	0.0069	0.43
	**NT-proBNP**							
Traditional assay	3.35	(1.63, 6.91)	0.0017	0.0068	2.65	(1.12, 6.28)	0.026	0.14
Aptamer, Raw	1.99	(1.25, 3.17)	0.0047	0.025	1.57	(0.90, 2.75)	0.11	0.88
Aptamer, Median Normalized	2.24	(1.28, 3.9)	0.0057	0.028	1.64	(0.83, 3.26)	0.15	0.51
Aptamer, ANML	2.21	(1.25, 3.92)	0.008	0.031	1.62	(0.80, 3.29)	0.17	0.52

Limma method was used to analyze fold change in 14 CERES participants who died vs. 26 who survived over median 2.5 years of follow-up. Aptamers are measured in ANML format unless otherwise noted. From among the proteins with FC significant at FDR<0.05, we chose the 10 largest and 10 smallest fold change for proteins measured with SomaScan. NT-proBNP was measured with traditional assay and SomaScan (we show FC using raw, median normalized, and ANML formats). Adjusted model includes age, history of stroke, systolic blood pressure, albumin, phosphorus, dialysis modality. Bold denotes associations that met the threshold of statistical significance after Bonferroni correction for 5000 proteins. SVEP1: Sushi, von Willebrand factor type A, EGF and pentraxin domain-containing protein 1.

We selected protein ‘hits’ from among those with fold change significant at FDR q<0.05 in ANML format, and then ranked associations by effect size. We show the 10 proteins with highest fold change and 10 proteins with lowest fold change in Table 3. Of the associations that met Bonferroni significance, two markers were higher in participants who died: SVEP1 (unadjusted fold change (95%CI) 2.14 (1.62, 2.82), p = 2.1x10-6; adjusted 1.44 (1.08, 1.92), p = 0.014) and keratocan (unadjusted fold change (95%CI) 1.74 (1.40, 2.15), p = 6.1x10-6; adjusted fold change 1.62 (1.22, 2.15), p = 0.0014). Eight markers were lower in participants who died, including (LANCL1), insulin growth factor 1, and insulin-like growth factor-binding protein 5. The 10 proteins with highest and lowest fold change in raw and median normalized formats are shown in **S2 Tables 9 and 10 in** S2 File.

## Discussion

We have conducted a quality control study of technical and biological variability of the large-scale proteomic assay, SomaScan V4.0, in plasma samples of patients with kidney failure. We observed excellent technical variability and very good orthogonal correlations. ANML data formatting reduces variability in paired samples, but has minimal impact on orthogonal correlations with traditional assays or the associations of proteins with the phenotype of mortality in this pilot study.

Overall, technical variability was low using any data format. Intra-assay CVs were excellent and consistent with prior studies in patients with non-dialysis dependent chronic kidney disease [21] or without chronic kidney disease [22]. Whether the analysis is technical or within-subject variability, it is clear that the same protein measured in two samples will have less variability in the ANML format. We observed lower spearman *rho* for short- and long-term biological variability, which is most likely because magnitude and direction of change in proteins over time is more heterogenous than for technical variability. We were able to utilize a small group (N = 4) of participants whose samples were used to measure both intra-assay and short-term within-subject variability. Historically, authors such as Fraser et al. have proposed that optimally for any given analyte, the (analytical CV) ≤ 0.5 (within-subject CV) [23]. We note that for 58% of the proteins, the analytical CV is less than ½ of the observed short-term within-subject variability, and for 92% the analytical CV < short-term within-subject CV (using ANML format). While these data are based on a modest number of samples, these estimates make us optimistic that individual proteins discovered with this platform could become viable biomarkers. We also tested whether proteins at the lowest concentrations had higher CVs, and found this was not the case. We would not recommend excluding protein measures based on lower limit of assay detection.

We observed very good orthogonal correlations (Spearman rho >0.7) for PTH, NT-proBNP, FGF-23 and CRP, but only moderate correlations for troponin T (*rho* = 0.64) and troponin I (*rho* = 0.51). Previously, a study of orthogonal correlations for three proteins using an earlier version of SomaScan, also showed a range of correlations [24]. While our results should be considered preliminary, it does suggest that an unexpected null finding for an established marker measured by aptamer could be a false negative if the aptamer assay for the marker is not highly correlated with the traditional assay.

Approximately 1% of proteins had concentrations too high to be quantified using routine SomaScan dilution protocols. The 47 proteins that saturated in >half of CERES samples are listed in **S2 Table 1 in** S2 File. It is interesting to note that a similar number of proteins saturate in plasma samples from patients with normal kidney function, although the saturating proteins may differ in these settings (unpublished data). Thus, the phenomenon may not be specific to samples from patients with kidney failure in whom concentrations of small molecular weight proteins are higher than normal populations. Since our ultimate goal is to develop biomarkers of CVD in patients with kidney failure, we reviewed whether these saturated proteins have potential roles in CVD. Alpha 1 microglobulin (A1M; 26kDa) is an anti-oxidant produced in the liver and freely filtered by the kidneys, and it is not surprising that this and other proteins of molecular weight <30 kDa are found on the list of saturated proteins in samples from patients with kidney failure. A1M plays a role in preventing oxidation of low density lipoprotein, (relevant to atherosclerosis) and may play a role in collagen metabolism in the extracellular matrix (relevant to left ventricular fibrosis and heart failure) [25]. Fatty acid-binding proteins (15 kDa) are lipid chaperones that may have roles in atherosclerosis, insulin resistance and heart failure [26,27]. Neurogranin (7–15 kDa) regulates endothelial nitric oxide synthase [28]. Guanosine 3’,5’-cyclic monophosphate -specific 3’,5’-cyclic phosphodiesterase, (100 kDa) may be present at high levels as a marker of CVD rather than as a physiological result of glomerular filtration [29]. Thus, while relatively few proteins saturate in the SomaScan assay, these and several other saturated proteins do have potential roles in CVD.

Several proteins differed between survivors and non-survivors at a Bonferroni level of statistical significance. At least half of deaths in kidney failure are typically due to CVD [30], and so it is not surprising that some of these are cardiovascular mediators. SVEP1 is an extracellular matrix protein. Epidemiological studies, human genetics and animal studies support its role in the development of atherosclerosis [31]. Keratocan maintains the extracellular matrix, and rare genetic variants have been associated with premature atherosclerosis [32]. Among those present at lower concentrations in participants who died, transketolase may promote cardiomyocyte apoptosis and play a role in ischemic heart failure [33]. Insulin-like growth factor I (IGF1) has been extensively researched and has pleiotropic effects in the myocardium and skeletal muscle. IGF1 is protective against atherosclerosis [34], and deletion of IGF1 receptors in mice results in dilated cardiomyopathy [35]. Insulin-like growth factor-binding protein 5 is part of IGF1 pathways. LANCL1 is a membrane protein that may have antimicrobial properties, and is protective against oxidative stress. Spinelli et al have proposed that LANCL1 may be cardioprotective in its role as a receptor for abscisic acid, a hormone that promotes nitric oxide production and glucose homeostasis [36].

### Limitations

The primary purpose of this quality control study was to establish the precision of the SomaScan assay among patients with kidney failure. While our study additionally examined and found biomarkers associated with mortality in kidney failure, a longer list is likely to evolve from our upcoming large study of kidney failure. We did not examine changes in the proteome resulting from each hemodialysis session itself, but we plan to conduct this type of study soon, using these within-subject variability analyses as a foundation for such analyses. The analysis of mortality was limited in sample size, increasing the likelihood of type II error and reducing the number of covariates that could be included in adjusted models.

## Conclusions

The SomaScan assay has excellent technical variability and low within-subject variability in plasma samples of study participants with kidney failure on dialysis. Comparing ANML and raw protein data, ANML format reduced variability in paired samples but had little impact on effect sizes in this population. Use of ANML formatting could facilitate comparison of biomarker results with other studies, most of which utilize this format. We expect SomaScan to provide novel and reproducible information in populations with kidney failure on dialysis.

## Supporting information

S1 File SomaScan technical information. (PDF)

S2 File S2 Table 1 Proteins Excluded From Variability Analyses. S2 Table 2 Baseline Characteristics of CRIC Participants with Kidney Failure on Hemodialysis. S2 Table 3 Proteins with Intra-assay CV>20% Using Raw or ANML Data Format. S2 Table 4 Proteins with Inter-assay CV>40% Using Raw or ANML Data Format. S2 Table 5 Short-term Biological Variability Measured Separately in 4 CERES Participants. S2 Table 6 Long-term Variablity of Proteins in 421 CRIC Participants: Raw Data Format. S2 Table 7 Long-term Variablity of Proteins in 421 CRIC Participants: ANML Data Format. S2 Table 8 Correlations of Fold Change and P-values of Proteins in Non-survivors. S2 Table 9 Fold Change (FC) in Protein Level in Non-Survivors Compared to Survivors Using Raw Data. S2 Table 10 Fold Change (FC) in Protein Level in Non-Survivors Compared to Survivors Using Median Normalized Data.
(ZIP)

## Abbreviations

ANML: Adaptive Normalization by Maximum Likelihood
CERES: Cardiac, Endothelial Function and Arterial Stiffness in End-Stage Renal Disease
CRIC: Chronic Renal Insufficiency Cohort
CRP: C-reactive protein
CV: Coefficient of variation
CVD: Cardiovascular disease
FGF-23: fibroblast growth factor 23
IQR: Interquartile range
LANCL1: LanC like glutathione S-transferase 1
NT-proBNP: N terminal pro-B-type natriuretic peptide
PTH: parathyroid hormone
RFU: Relative fluorescence unit
SD: Standard deviation
SVEP1: Sushi, von Willebrand factor type A, EGF and pentraxin domain containing 1
UCSF: University of California San Francisco
