# Supplementary material for: Evaluation of a large-scale aptamer proteomics platform among patients with kidney failure on dialysis
Source: PLoS One. 2023 Dec 11;18(12):e0293945. doi: 10.1371/journal.pone.0293945 (PMC10712847; doi:10.1371/journal.pone.0293945)
Supplement: S1 File — (PDF) [file pone.0293945.s001.pdf]

SomaScan® Assay v4.0 Technical Note

The SomaScan Platform is a powerful tool with applications in scientific research and health and wellness.

This technical note provides background on the platform itself, as well as performance metrics for the SomaScan Assay v4.0.

SomaLogic® SomaScan® SOMAmer® and associated logo are registered trademarks of SomaLogic, Inc. and any third party trademarks used herein are the property of their respective owners
© 2021 SomaLogic, Inc. | 2945 Wilderness Pl. Boulder, CO 80301 | Ph 303 625 9000 | www.somalogic.com
Republished from Somalogic Technical Note under a CC BY license, with permission from Somalogic, original copyright 2021.

Introduction

The SomaScan Assay from SomaLogic is the only proteomic technology capable of measuring rapidly (high throughput), broadly (thousands of proteins simultaneously), and deeply (high-and low-abundance proteins).

The SomaScan Platform is a highly multiplexed, sensitive, quantitative, and reproducible proteomic tool for discovering previously unidentified biomarkers for drug discovery, pre-clinical and clinical drug development, and clinical diagnostics, across a wide range of important diseases and conditions.

The SomaScan Assay v4.0 measures approximately 5,000 unique human protein analytes in small volumes of biological samples. The assay was developed for performance in human serum and plasma and offers exceptional dynamic range, quantifying the relative levels of proteins in plasma that span 10 logs in abundance with excellent reproducibility. Recently human urine was added to the standard list of matrices for the SomaScan Platform.

The SomaScan Platform is enabled by the generation of protein-capture reagents called SOMAmer® (Slow Off-rate Modified Aptamer) reagents. SOMAmer reagents consist of short single-stranded DNA sequences that incorporate hydrophobic modifications, greatly expanding the physicochemical diversity of the large randomized nucleic acid libraries from which the SOMAmer reagents are selected.

The SomaScan Platform measures native proteins in complex matrices by transforming available binding sites on individual proteins into a corresponding SOMAmer reagent concentration, which is then quantified by hybridization to microarrays.

The assay takes advantage of SOMAmer reagents' dual nature as both protein affinity-binding reagents with defined three-dimensional structures and unique nucleotide sequences recognizable by specific DNA hybridization probes.

SomaScan Assay data are used to build models called SomaSignal™ Tests. The SomaScan Assay and SomaSignal Tests were developed and are performed in our laboratory in compliance with Clinical Laboratory Improvement Amendments (CLIA) standards for Laboratory Developed Tests (LDT). The SomaLogic laboratory is registered with the Centers for Medicare & Medicaid Services (CMS) under CLIA of 1988 and is accredited by the College of American Pathologists (CAP).

The SomaScan Platform is being applied to a wide range of diseases and conditions to deliver insights that enable biomarker discovery, diagnostics development, pharmaceutical discovery and development, and health management.

© 2021 SomaLogic, Inc. | 2945 Wilderness Pl. Boulder, CO 80301 | Ph 303 625 9000 | www.somalogic.com
Republished from Somalogic Technical Note under a CC BY license, with permission from Somalogic, originally copyright 2021

Previous versions of the SomaScan Assay have been applied successfully to biomarker discovery and validation in many pharmaceutical research and development projects, diagnostics discovery and development projects and academic research projects. A selected list of peer-reviewed articles can be found here.

SOMAmer reagents are discovered using robust SELEX technology

SOMAmer reagents are single stranded DNA-based protein affinity reagents that benefit from aptamer technology developed over the past 30 years[1,2]. The more recent proprietary innovation incorporates chemically modified nucleotides (Figure 1) that facilitate the binding to proteins, expanding the chemical diversity of standard aptamers and enhancing the specificity and affinity of protein-nucleic acid interactions[3].

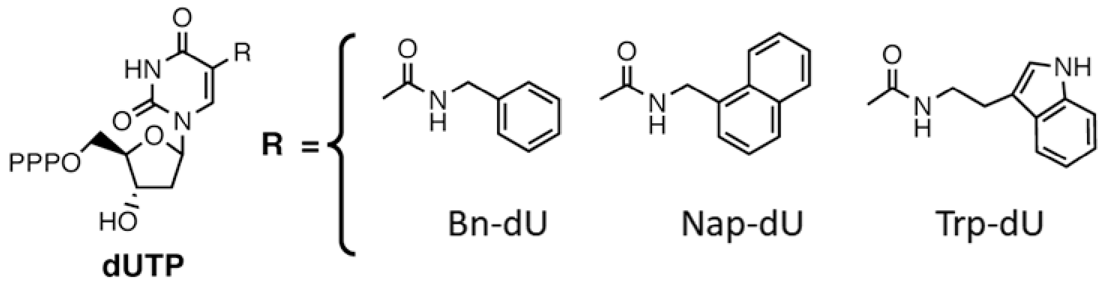

Figure 1. Modified nucleotides. Nucleotide triphosphate analogs modified at the 5-position (R) of uridine; 5-benzylaminocarbonyl-dU; 5-naphthylmethylaminocarbonyl-dU; 5-tryptaminocarbonyl-dU

These modified nucleotides are incorporated into nucleic acid libraries used for the iterative selection and amplification process called SELEX (Systematic Evolution of Ligands by EXponential enrichment) from which SOMAmer reagents are selected[4–6]. Using a novel, proprietary SELEX process, SomaLogic has generated SOMAmer reagents to proteins that had been resistant to selection with unmodified nucleic acids (ACTG traditional aptamers)[3].

These chemical modifications in turn allow the SOMAmer reagent to tightly bind its target protein in ways never before possible[7]. A key advantage of this artificial selection process is that conditions can be tailored to select for desirable properties, including specificity, slow off-rate, and specific assay conditions.

SOMAmer reagents are selected against proteins in their native folded conformations and are therefore generally found to require an intact, tertiary protein structure for binding.

© 2021 SomaLogic, Inc. | 2945 Wilderness Pl. Boulder, CO 80301 | Ph 303 625 9000 | www.somalogic.com
Republished from Somalogic Technical Note under a CC BY license, with permission from Somalogic, originally copyright 2021

As such, unfolded and denatured—and therefore presumably inactive—proteins are not detected by SOMAmer reagents. Crystal structures of SOMAmer reagents bound to their cognate protein targets indicate that the modified nucleotides contribute extensively to intramolecular contacts within the SOMAmer reagent and to intermolecular contacts with the protein targets[6].

In Figure 2, the X-ray crystal structure of the PDGF-BB SOMAmer reagent bound to PDGF-BB demonstrates that the interactions between the SOMAmer reagent and its cognate protein are mainly mediated via the modified nucleotides. The co-crystal structures show very specific interactions between the SOMAmer reagent and target with binding site dimensions of 1100-1200 $Å^2$, similar to antibody–antigen interactions[8].

The dissociation kinetics of a subset of SOMAmer reagents binding to their respective targets were determined using a solution-phase radiolabeled binding assay[3], and a subset were confirmed using the Biacore Flexchip surface plasmon resonance biosensor.

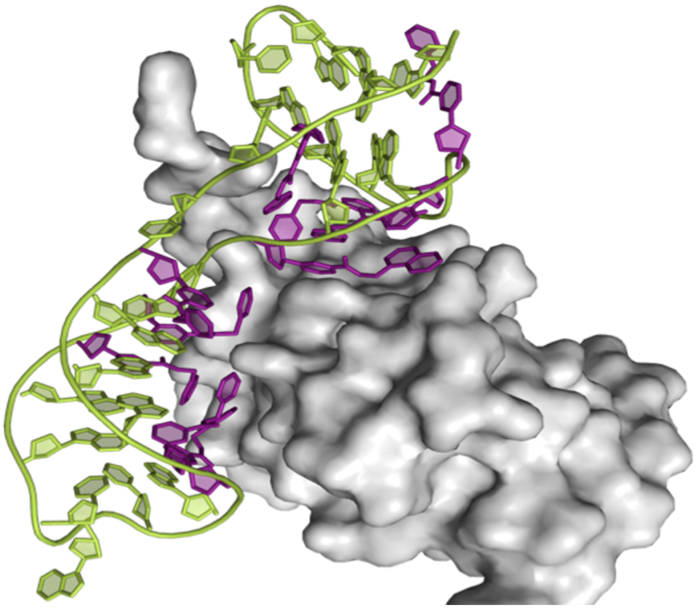

Figure 2. X-ray crystal structure of a SOMAmer reagent bound to PDGF-BB. Modified nucleotides are shown in purple, and the DNA backbone and unmodified bases are in green.

Biosensor results confirm slow dissociation off-rates, in the 10^{-5} to 10^{-4} s^{-1} range, that correlate well with dissociation rate constants measured by solution-phase radiolabeled binding assays. Overall, SOMAmer reagents are analogous to high-quality antibodies that recognize intact tertiary protein structures.

© 2021 SomaLogic, Inc. | 2945 Wilderness Pl. Boulder, CO 80301 | Ph 303 625 9000 | www.somalogic.com
Republished from Somalogic Technical Note under a CC BY license, with permission from Somalogic, original copyright 2021

However, since they are made out of nucleic acids, SOMAmer reagents have several advantages over antibodies, such as tailored in vitro selection conditions, chemical synthesis, storage stability, and detection using sensitive and advanced DNA detection methods.

SOMAmer Reagents to Approximately 5,000 Human Proteins

SOMAmer reagents have been created for human protein targets that cover a diverse set of biological processes, including cancer, inflammation and cardiovascular function, to name a few. Targets to date extensively cover major molecular functions including receptors, kinases, growth factors and hormones, and span a diverse collection of secreted, intracellular and extracellular proteins or domains.

SOMAmer Reagents are Chemically Synthesized, Stable and Rigorously Analyzed

After identification using SELEX technology, the SOMAmer reagents are chemically synthesized, purified and analyzed by methods including ultra high-performance liquid chromatography (UPLC), capillary gel electrophoresis (CGE) and mass spectrometry (MS). Extensive functional analysis ensures consistent high performance of the SOMAmer reagents as quantitative affinity reagents. In order to test the specificity of SOMAmer reagents used in the SomaScan Assay for their respective initial target proteins, we perform a variety of characterization steps.

These steps include:

- In silico selection, procurement (when available), and direct SOMAmer reagent binding experiments in buffer with related proteins.

- Pull-down assays followed by MS-based and SDS gel-based analyses of the protein(s) bound by the SOMAmer reagent from biological matrices.

All types of affinity reagents (antibodies, traditional aptamers, etc.) are subject to specificity issues. Recognizing how critical the accuracy of the SomaScan Assay is for both research and clinical purposes, SomaLogic is committed to regular assessment of SOMAmer reagent specificity, and to transparency in communicating the results of those ongoing efforts.

Additional information can be found in The Characterization of SOMAmer Reagents Technical Note (SL00000430).

The Assay Steps

The SomaScan Assay quantitatively transforms the protein epitope availability in a biological sample into a specific SOMAmer-based DNA signal (Figure 3).

© 2021 SomaLogic, Inc. | 2945 Wilderness Pl. Boulder, CO 80301 | Ph 303 625 9000 | www.somalogic.com
Republished from Somalogic Technical Note under a CC BY license, with permission from Somalogic, original copyright 2021

A SOMAmer-protein binding step is followed by a series of partitioning and wash steps to convert relative epitope concentrations into measurable nucleic acid signals that are quantified using DNA-hybridization microarrays. Assay details are provided in the Appendix and in Gold et al., 2010[3]. The readout in relative fluorescent units (RFU) is directly proportional to the amount of target epitope in the initial sample.

Achieving the 10^{10} Dynamic Range: SOMAmer Reagent Mixes

The large dynamic range of the SomaScan Assay in plasma and serum results from the optimization of SOMAmer reagent measurements across three serial dilutions of the sample (Figure 4). A specific SOMAmer reagent is only present in one of the three dilution groups. The least concentrated sample is designed to detect the most abundant proteins, and the most concentrated solution is designed to detect the least abundant proteins. Based on reported literature values, the SomaScan Assay robustly measures analytes spanning a range of 10 logs in human plasma or serum (e.g. albumin to interleukins or interferons).

SomaScan Assay Characterization

The SomaScan Assay v4.0 has been validated in human EDTA plasma, serum, and urine. The SomaScan Assay has excellent reproducibility. In plasma, half of the SOMAmer reagents demonstrated a median Coefficient of Variation (CV) less than 5.0% and only 10% of the SOMAmer reagents demonstrated a median CV of 11.8% or higher. All values were determined in the multiplex assay, profiling approximately 5,000 reagents simultaneously. SomaScan Assay metrics using urine can be found in our Urine Measurements on the SomaScan Platform Technical Note (SL00000472).

By measuring thousands of proteins at a time in each blood sample with the SomaScan Platform, it is possible to uncover a precise set of specific protein changes that provide information on current status and future trajectory for virtually every disease or condition of interest. For example, we can find repeatable patterns of protein changes that are associated with clinical indications (e.g., cardiovascular events) or fitness attributes (e.g., VO_2 Max).

Each set of specific protein patterns, in turn, becomes the basis of a specific SomaSignal Test that can be ordered by researchers who desire clinical assessments of study participants. The current suite of available RUO SomaSignal Tests can be found on the SomaLogic website.

SomaLogic Quality Systems

The SomaScan Assay is performed under the SomaLogic Quality System (QS) in a laboratory that follows CLIA standards for a laboratory developed test.

© 2021 SomaLogic, Inc. | 2945 Wilderness Pl. Boulder, CO 80301 | Ph 303 625 9000 | www.somalogic.com
Republished from Somalogic Technical Note under a CC BY license, with permission from Somalogic, original copyright 2021

The assay is performed in a facility that contains both access and environmental control. Equipment within the facility is maintained, calibrated, and operated in compliance with controlling Standard Operating Procedures (SOPs). Equipment and associated software are validated for their intended uses in support of the SomaScan Assay.

Method validation has been completed for processes that could impact the performance of the SomaScan Assay. SOPs cover the incoming receipt, inspection, and release of raw materials to ensure that the materials used in the production of assay reagents or directly in the assay maintain the performance requirements established during the development of the SomaScan Assay.

Step 1
SOMAmer reagents (purple) are synthesized with a fluorophore, photocleavable linker, and biotin.

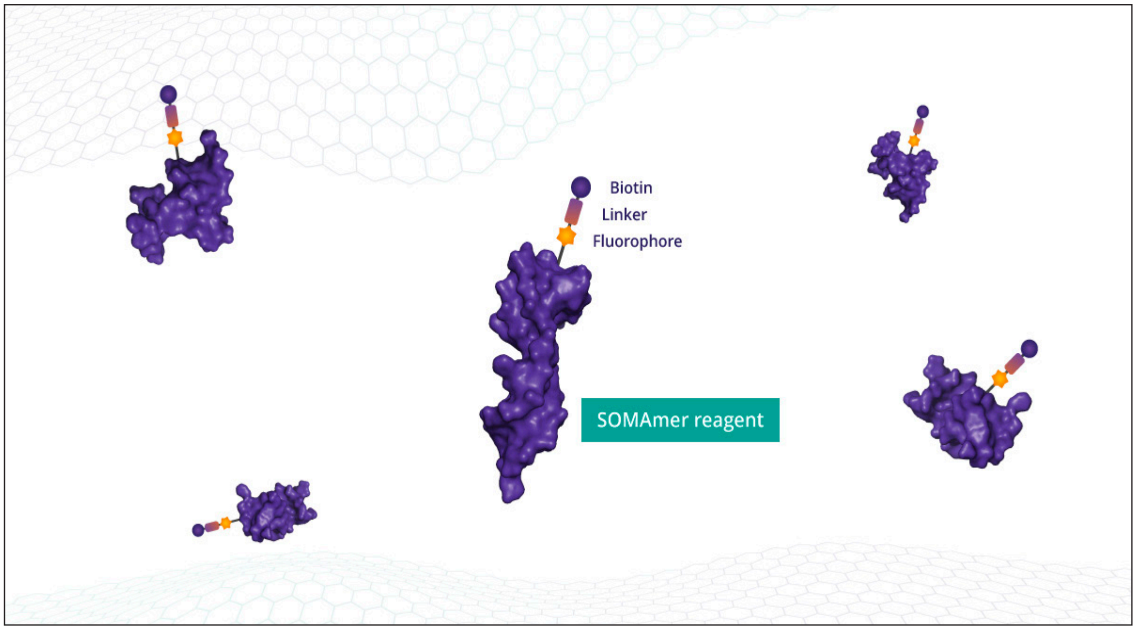

Step 2
SOMAmer reagents bound to streptavidin beads are used to capture proteins from a complex mixture of proteins (tan).

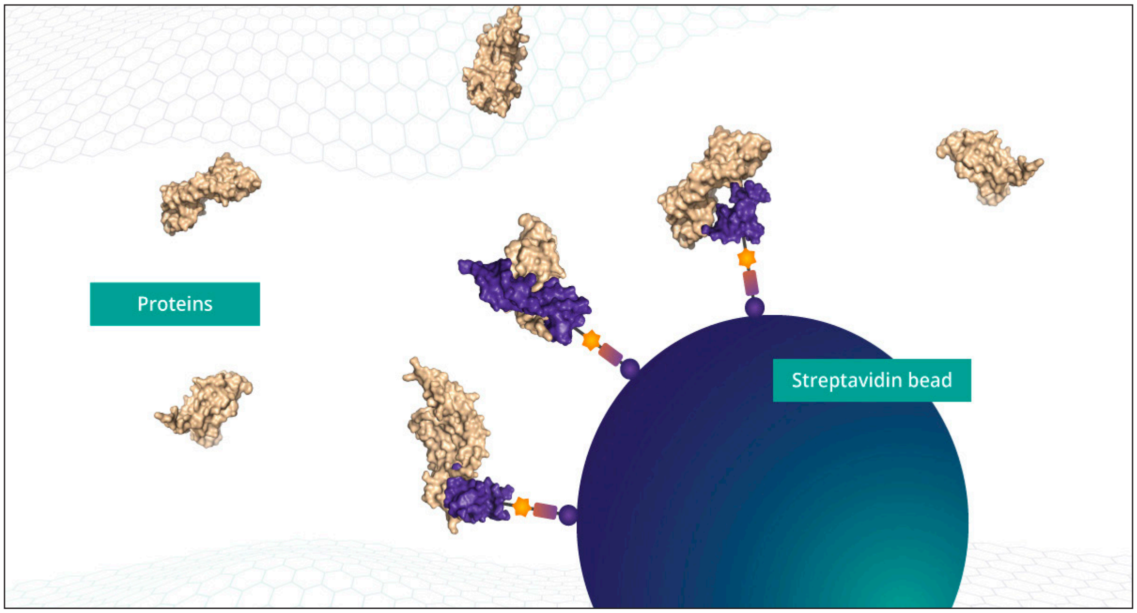

© 2021 SomaLogic, Inc. | 2945 Wilderness Pl. Boulder, CO 80301 | Ph 303 625 9000 | www.somalogic.com
Republished from Somalogic Technical Note under a CC BY license, with permission from Somalogic, original copyright 2021

Step 3
Unbound proteins are washed away, and bound proteins are tagged with biotin.

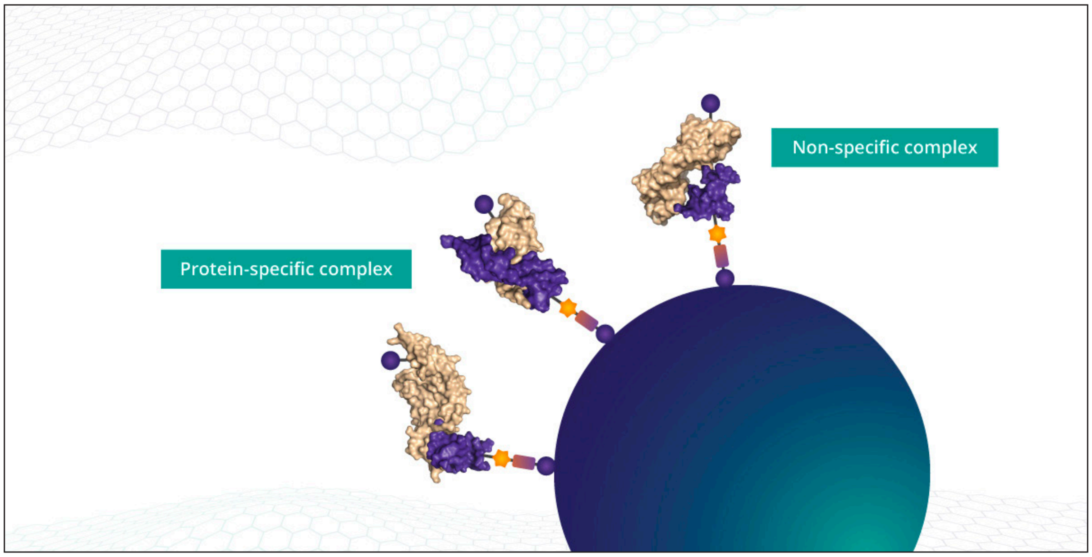

Step 4
UV light breaks the photocleavable linker, releasing complexes back into solution.

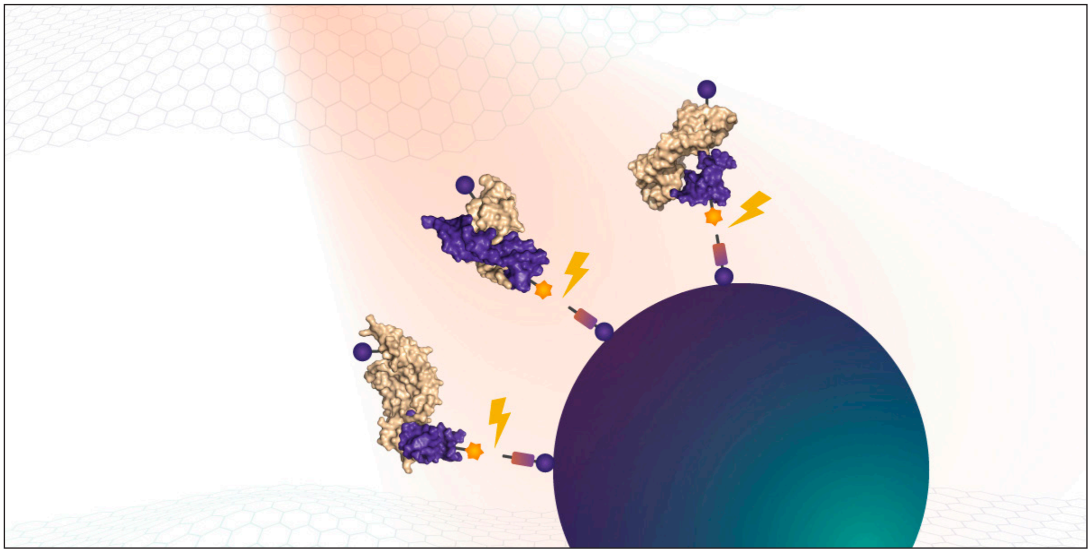

Step 5
Non-specific complexes dissociate while specific complexes remain bound.

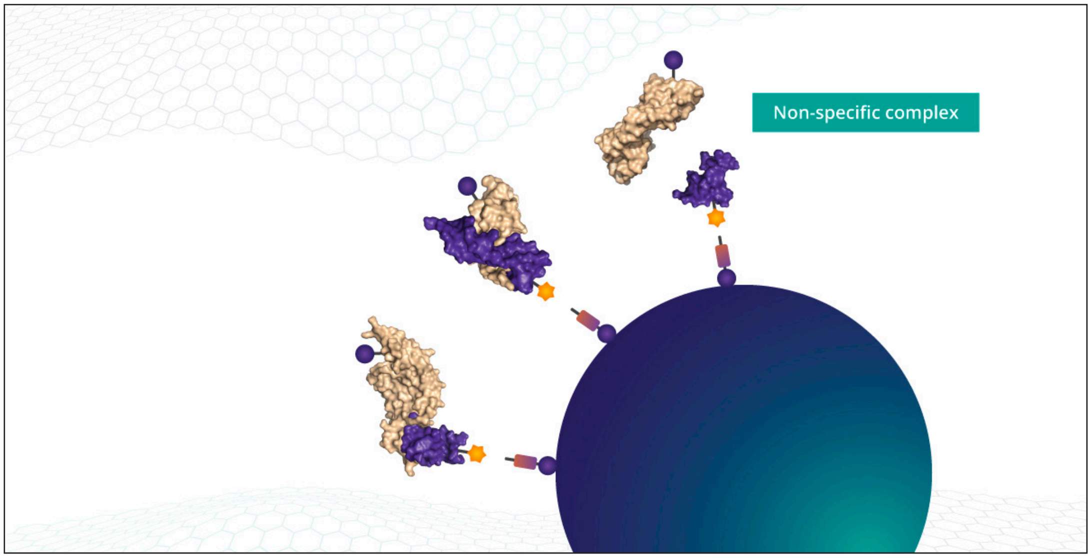

© 2021 SomaLogic, Inc. | 2945 Wilderness Pl. Boulder, CO 80301 | Ph 303 625 9000 | www.somalogic.com
Republished from Somalogic Technical Note under a CC BY license, with permission from Somalogic, original copyright 2021

Step 6
A polyanionic competitor (green) prevents rebinding of non-specific complexes.

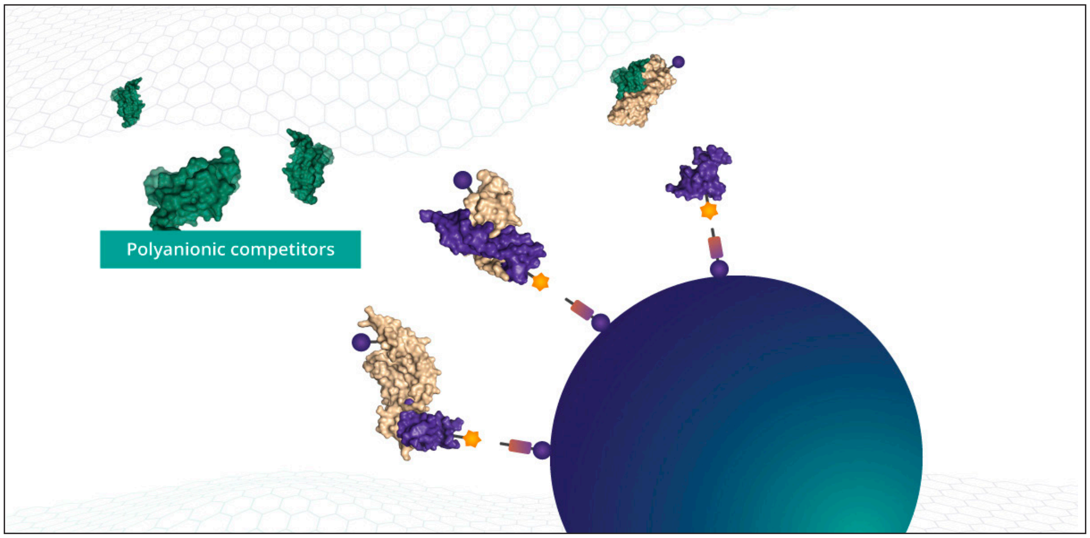

Step 7
Biotinylated proteins (and bound SOMAmer reagents) are captured on new streptavidin beads.

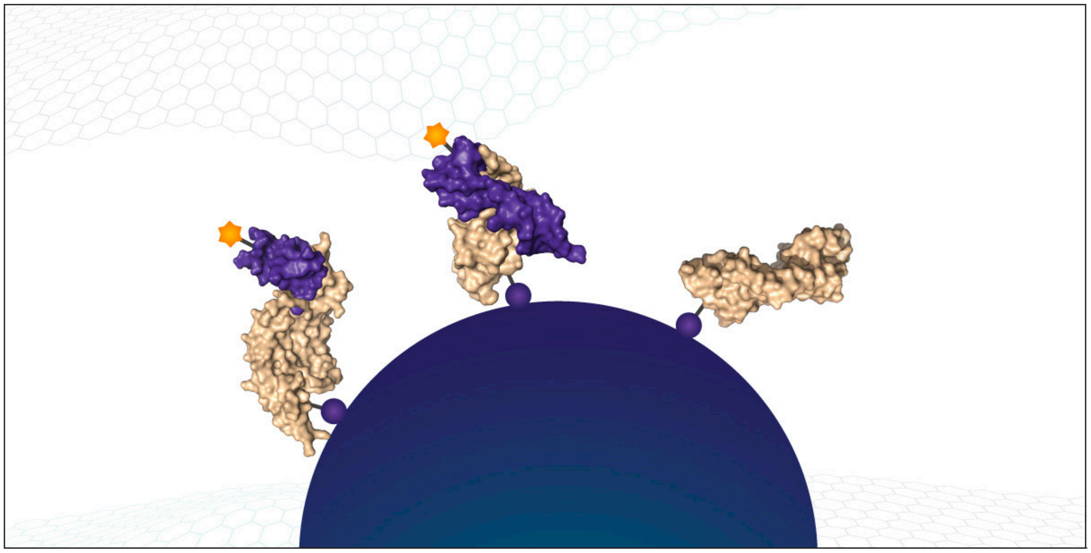

Step 8
SOMAmer reagents are released from the complexes by denaturing the proteins. Fluorophores are measured after hybridization to complementary sequences on a microarray chip. The fluorescence intensity detected on the microarray is related to the amount of available epitope in the original sample.

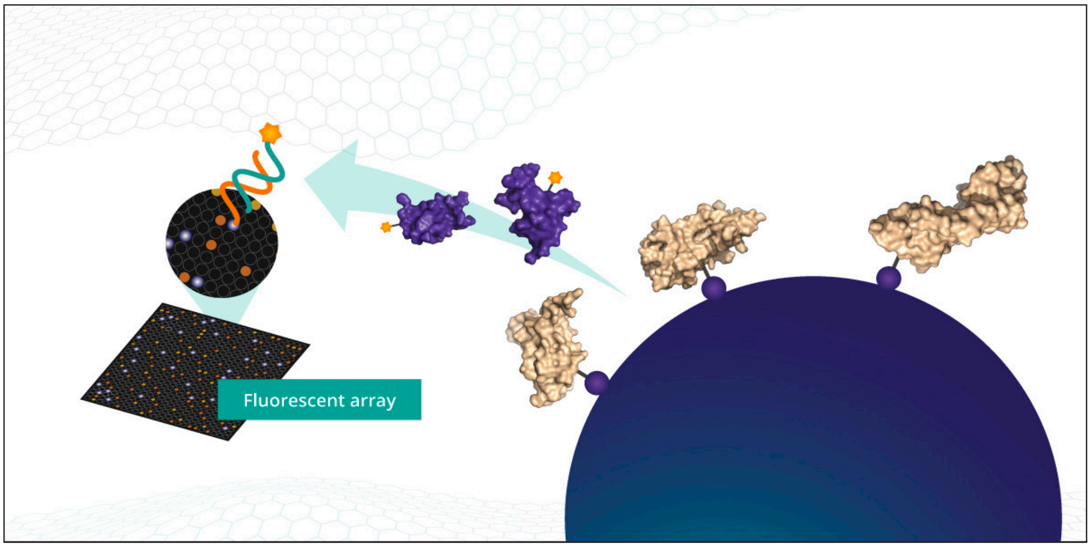

© 2021 SomaLogic, Inc. | 2945 Wilderness Pl. Boulder, CO 80301 | Ph 303 625 9000 | www.somalogic.com
Republished from Somalogic Technical Note under a CC BY license, with permission from Somalogic, original copyright 2021

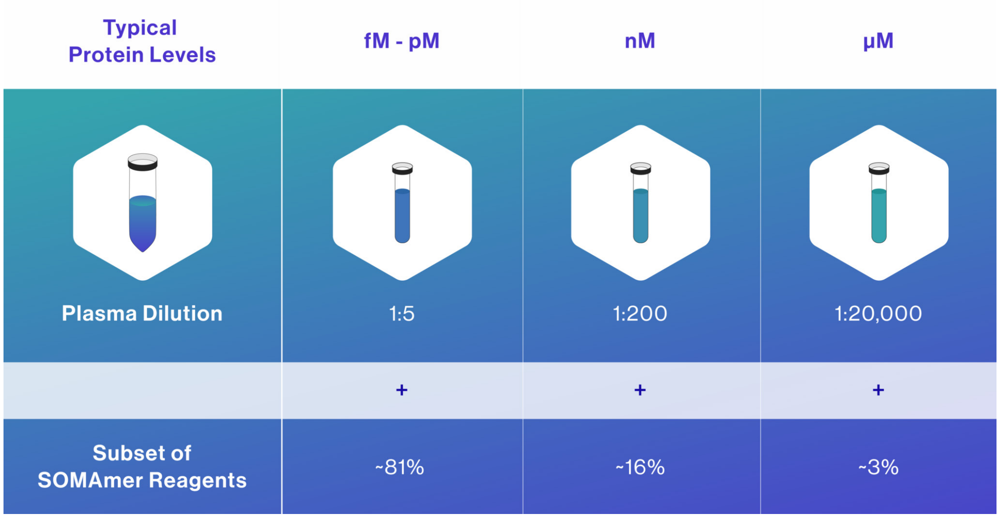

Figure 4. SomaScan Assay dynamic range. SOMAmer reagent mixes are prepared to achieve optimal detection in mixtures with a large range of concentrations. Shown here is the dilution distribution of SOMAmer reagents for SomaScan Assay v4.0 with plasma and serum.

Summary

The SomaScan Platform is a powerful, highly multiplexed platform for discovering novel biomarkers during drug discovery, pre-clinical and clinical drug development, and for the development of clinical diagnostics, across a wide range of clinically important diseases.

Table 1 summarizes the SomaScan Assay v4.0 metrics in plasma. SomaLogic's SomaScan Platform technology provides significant advantages in sample size, cost, time, multiplexing capability, measurement range, and flexibility of readout over many alternate protein biomarker platforms.

Metric	Condition	Results
Sensitivity in Buffer	Median LOD (from representative subset)	100 fM or 4.2 pg/mL
Measurement Range	Span of proteins measured in plasma or serum	10^{10}
Precision	Median Total % CV in plasma	5%
Sample Volume	Human serum or plasma (per sample)	130 µL
Multiplex Size	Total number of measurements	5,284
	SOMAmer reagent measurements	4,979

Table 1. Summary of SomaScan v4.0 Assay metrics to human targets.

© 2021 SomaLogic, Inc. | 2945 Wilderness Pl. Boulder, CO 80301 | Ph 303 625 9000 | www.somalogic.com
Republished from Somalogic Technical Note under a CC BY license, with permission from Somalogic, original copyright 2021

References

1. Ellington AD & Szostak JW. (1990). In vitro selection of RNA molecules that bind specific ligands. Nature 346:818-22.
2. Tuerk C & Gold L. (1990). Systematic evolution of ligands by exponential enrichment: RNA ligands to bacteriophage T4 DNA polymerase. Science 249:505-10.
3. Gold L, *et al.* (2010). Aptamer-based multiplexed proteomic technology for biomarker discovery. PLoS One 5(12): e15004.
4. Vaught JD, et al. (2010). Expanding the chemistry of DNA for in vitro selection. J. Am. Chem. Soc. 132:4141-51.
5. Eaton BE. (1997). The joys of in vitro selection: chemically dressing oligonucleotides to satiate protein targets. Curr. Opin. Chem. Biol. 1:10-6.
6. Davies DR, et al. (2012). Unique motifs and hydrophobic interactions shape the binding of modified DNA ligands to protein targets. Proc. Natl. Acad. Sci. USA 109:19971-6.
7. Rohloff J, et al. (2014). Nucleic acid ligands with protein-like side chains: Modified aptamers and their use as diagnostic and therapeutic agents. Mol. Ther. Nucleic Acids 3: e201.
8. Ramaraj T, et al. (2012). Antigen-antibody interface properties: Composition, residue interactions, and features of 53 non-redundant structures. Biochim. Biophys. Acta. 1824:530-2.

© 2021 SomaLogic, Inc. | 2945 Wilderness Pl. Boulder, CO 80301 | Ph 303 625 9000 | www.somalogic.com
Republished from Somalogic Technical Note under a CC BY license, with permission from Somalogic, original copyright 2021

Appendix

Details of the SomaScan Assay

The first step of the SomaScan Assay is the dilution of a biological sample of interest. The sample dilutions are incubated with the respective SOMAmer reagent mixes that have been attached to streptavidin (SA)-coated beads.

The beads are washed to remove non-specifically associated proteins and other matrix constituents. Proteins that remain bound to SOMAmer reagents are tagged using an NHS-biotin reagent. SOMAmer complexes and unbound SOMAmer reagents are released from the SA beads using ultraviolet light that cleaves a photo-cleavable linker within the SOMAmer reagent construct into a solution containing an anionic competitor.

Non-specific interactions dissociate, and the anionic competitor solution prevents them from reforming while specific complexes are maintained[3]. The photo-cleavage eluate, which contains all SOMAmer reagents (some bound to a biotin-labeled protein and some free), is separated from the beads and then incubated with a second streptavidin coated bead that binds the biotin-labeled proteins and the biotin-labeled protein–SOMAmer complexes.

The free SOMAmer reagents are removed during subsequent washing steps. In the final elution step, protein-bound SOMAmer reagents are released from their proteins using denaturing conditions and recovered. These SOMAmer reagents are then quantified by hybridization to custom DNA microarrays. The cyanine-3 signal from the SOMAmer reagent is detected on microarrays.

Data Methods

Experimental Controls for Data Standardization and Quality Control Processing

The SomaScan Assay is performed using 96-well plates; eleven wells are allocated for control samples used to control for batch effects and to estimate the accuracy, precision, and buffer background levels of the assay. Five pooled Calibrator Control replicates, three pooled Quality Control (QC) replicates, and three buffer (no protein) replicates are run on each plate.

For core sample types, Calibrator and QC replicates are created by pooling samples of the same type from presumed healthy donors. Twelve Hybridization Control SOMAmer reagents not exposed to sample proteins are added during the SOMAmer reagent elution step to control for readout variability.

© 2021 SomaLogic, Inc. | 2945 Wilderness Pl. Boulder, CO 80301 | Ph 303 625 9000 | www.somalogic.com
Republished from Somalogic Technical Note under a CC BY license, with permission from Somalogic, original copyright 2021

Appendix

The control samples are run repeatedly during assay qualification and robust point estimates are generated and stored as references for each SOMAmer reagent result for the Calibrator and QC samples. The results are to be used as references throughout the life of the SomaScan Assay version.

Data Standardization

Raw SomaScan Assay data may contain systematic biases from many sources, such as technical variation introduced by the readout, pipetting errors, or consumable reagent changes; more significantly, pre-analytical variance related to sample collection methods or inherent sample variation in overall protein levels leads to additional nuisance variance. Data standardization procedures are used to mitigate technical variation.

Data standardization is comprised of normalization and calibration which are routine numerical procedures developed to remove systematic biases in raw assay data after microarray feature aggregation.

In general, normalization is a sample-by-sample adjustment to overall signals within dilution bins (plasma and serum) or signaling bins (urine). Calibration is an overall plate adjustment and a SOMAmer-by-SOMAmer adjustment that decreases between-plate variability.

Each normalization method computes a scale factor, or set of scale factors, for each sample or SOMAmer reagent that is subsequently applied to the signal on the appropriate results in the report.

The data standardization steps include the following:

- Hybridization normalization
- Intra plate signal normalization of Calibrator and Buffer (no protein) replicates
- Plate scale standardization and Calibration using a global calibrator reference
- Signal normalization of the QC replicates using a global signal normalization reference
- QC check of the median of QC replicate values to the global QC reference standard specific for the pooled QC lot on the plate
- Signal normalization of the individual samples using a global signal normalization reference

For more details on the data standardization process see document our Data Standardization and File Specification Technical Note (SL00000442).

© 2021 SomaLogic, Inc. | 2945 Wilderness Pl. Boulder, CO 80301 | Ph 303 625 9000 | www.somalogic.com
Republished from Somalogic Technical Note under a CC BY license, with permission from Somalogic, original copyright 2021

SomaLogic

Short Note

SomaScan® v4 Data Standardization

June 2020

SomaLogic®, SOMAmer®, SomaScan® and associated logo are registered trademarks of SomaLogic, Inc.

© 2020 SomaLogic, Inc. | 2945 Wilderness Pl. Boulder, CO 80301 | www.somalogic.com | SL00000442R2

Republished from Somalogic Technical Note under a CC BY license, with permission from Somalogic, original copyright 2021

Raw SomaScan Assay data may contain systematic biases from many sources, such as variation introduced by the readout, pipetting errors, inherent sample variation, or consumable reagent changes. Standardization is an important tool for removing nuisance variance.

This document briefly explains five techniques used to standardize SomaScan v4 Assay results and a sixth quality control step.

A schematic of the SomaScan Assay is illustrated below with colored boxes indicating the source of variation eliminated by each data standardization technique:

1. **Hybridization normalization** controls for variability in the readout of individual microarrays using 12 control SOMAmer® reagents added to the eluate just prior to readout.
2. **Intraplate median signal normalization** controls for total signal differences caused by assay variance between individual Calibrator Controls within an assay run.
3. **Plate scaling** accounts for plate-by-plate total signal variation typically associated with scanner intensity differences.
4. **Calibration** removes the variation between assay runs within and across experiments on a SOMAmer-by-SOMAmer reagent basis.
5. **Adaptive normalization to a reference** controls for total signal differences between individual samples and is an optional step that is dependent on the study design and expectations.

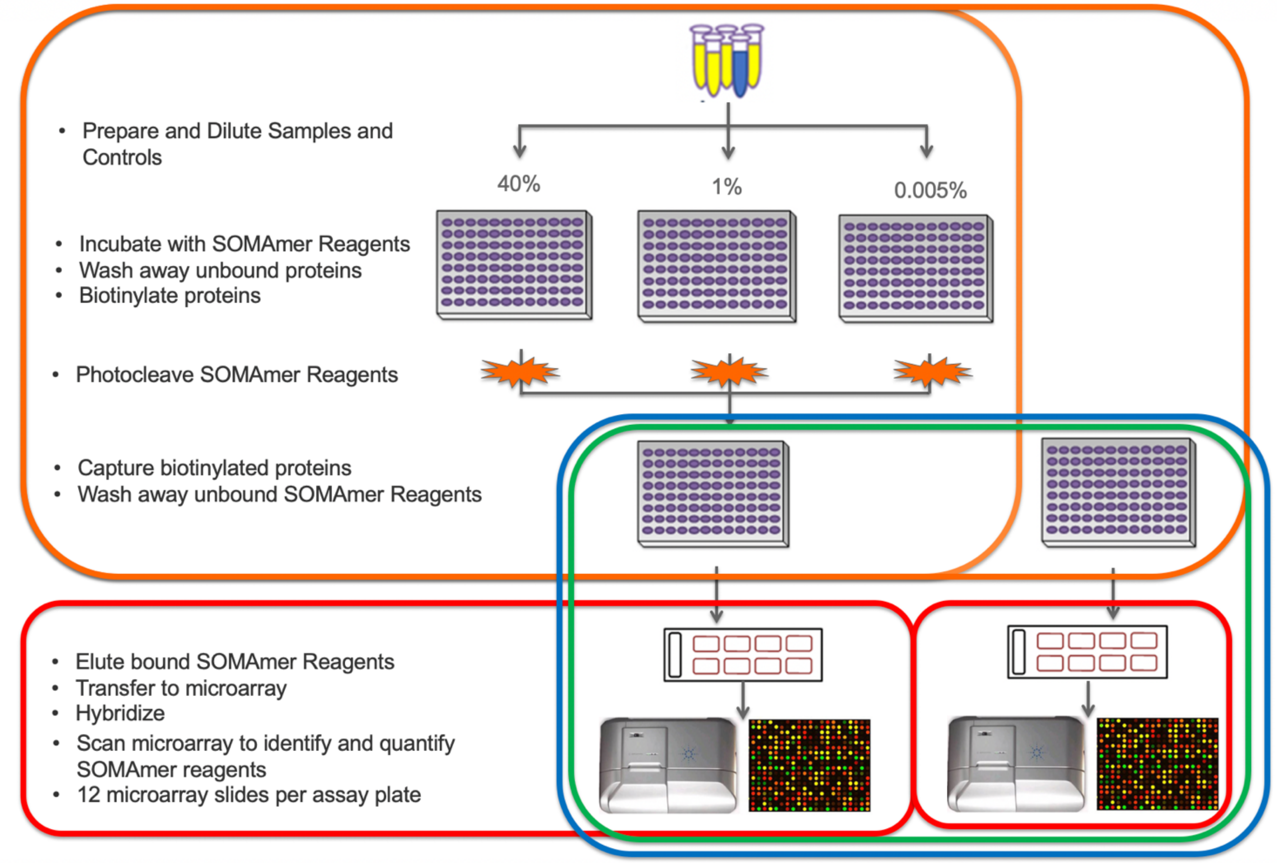

© 2020 SomaLogic, Inc. |2945 Wilderness Pl. Boulder, CO 80301 | www.somalogic.com | SL00000442R2

Republished from Somalogic Technical Note under a CC BY license, with permission from Somalogic, original copyright 2021

6. **Quality control check** uses replicate Quality Controls (QC) that are pooled matrix-matched samples (e.g. plasma, serum) run in the SomaScan Assay alongside clinical samples to quantify the quality of each assay run by determining the accuracy of the median replicate signal for each SOMAmer reagent compared to the reference. QC check is performed after steps 1-5 above have been applied.

Hybridization normalization mitigates bias caused by differential readout conditions (eluate transfer, hybridization, wash, scan) in individual microarrays. Control SOMAmer reagents are not exposed to protein but added during the transfer of SOMAmer reagents to the Agilent slide. The control signals are then used to calculate a single scaling factor that is applied to all spots in the microarray.

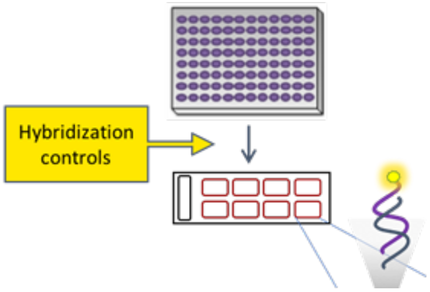

Intraplate median signal normalization was developed to account for differences in total signal between replicate control samples. It uses measurements from Calibrator Controls to calculate a sample-based scaling factor for each of the three dilution groups.

Like the Quality Controls, the Calibrator Controls are matrix-matched samples (e.g. plasma, serum) that are run in replicate in the SomaScan Assay alongside clinical samples. Plate scaling and calibration adjustments require data from invariant controls that are run in replicate in all assays. These data are used in conjunction with references generated during assay qualification to calculate a scaling factor for every plate and SOMAmer reagent.

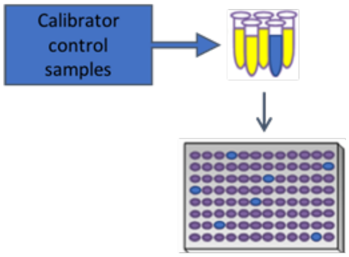

Plate scaling adjusts for total signal variation that occurs from plate to plate, typically associated with scanner intensity differences. The median value of the calibrator reference ratios is used to calculate a single scale factor for an entire plate.

Calibration reduces the variability between runs and/or entire experiments on a SOMAmer-by-SOMAmer reagent basis. The ratio of the SOMAmer-specific reference value to the median value of the Calibrator Controls is the calibration scale factor for the SOMAmer reagent across the run.

Adaptive normalization to a reference, Adaptive Normalization by Maximum Likelihood (ANML), adjusts for inter-sample technical and biological variability in total signal within and between runs. A scale factor for each set of SOMAmer reagents within a dilution bin is computed using only sample values within two

© 2020 SomaLogic, Inc. |2945 Wilderness Pl. Boulder, CO 80301 | www.somalogic.com | SL00000442R2

Republished from Somalogic Technical Note under a CC BY license, with permission from Somalogic, original copyright 2021

population standard deviations of the normal reference; the process is iterated to convergence and maximizes the probability that a sample's RFU measurements come from the reference distribution. The scale factor for each dilution bin is then applied to their respective SOMAmer reagents. This step is applied to QC and individual samples.

***Note**: Normalization to a reference standardizes the overall signal from every sample and is appropriate for experiments where all samples should have approximately the same amount of total protein.*

For samples with inherently different protein amounts (e.g. Duchenne Muscular Dystrophy or leukemic blood), median signal normalization to a reference will mute variations in protein levels that may be of interest. For further details, please refer to the SomaLogic Technical Note SL00000063.

SomaLogic®, SOMAmer®, SomaScan® and associated logo are registered trademarks of SomaLogic, Inc.

© 2020 SomaLogic, Inc. |2945 Wilderness Pl. Boulder, CO 80301 | www.somalogic.com | SL00000442R2

Republished from Somalogic Technical Note under a CC BY license, with permission from Somalogic, original copyright 2021
